# Nitarsone, Inorganic Arsenic, and Other Arsenic Species in Turkey Meat: Exposure and Risk Assessment Based on a 2014 U.S. Market Basket Sample

**DOI:** 10.1289/EHP225

**Published:** 2016-10-13

**Authors:** Keeve E. Nachman, David C. Love, Patrick A. Baron, Anne E. Nigra, Manuela Murko, Georg Raber, Kevin A. Francesconi, Ana Navas-Acien

**Affiliations:** 1 Johns Hopkins Center for a Livable Future,; 2 Department of Environmental Health Sciences,; 3 Department of Health Policy and Management,; 4 Risk Sciences and Public Policy Institute, and; 5 Department of Epidemiology, Johns Hopkins Bloomberg School of Public Health, Baltimore, Maryland, USA; 6 Department of Environmental Health Sciences, Columbia University Mailman School of Public Health, New York, New York, USA; 7 Institute of Chemistry-Analytical Chemistry, University of Graz, Graz, Austria

## Abstract

**Background:**

Use of nitarsone, an arsenic-based poultry drug, may result in dietary exposures to inorganic arsenic (iAs) and other arsenic species. Nitarsone was withdrawn from the U.S. market in 2015, but its use in other countries may continue.

**Objectives:**

We characterized the impact of nitarsone use on arsenic species in turkey meat and arsenic exposures among turkey consumers, and we estimated cancer risk increases from consuming turkey treated with nitarsone before its 2015 U.S. withdrawal.

**Methods:**

Turkey from three cities was analyzed for total arsenic, iAs, methylarsonate (MA), dimethylarsinate, and nitarsone, which were compared across label type and month of purchase. Turkey consumption was estimated from NHANES data to estimate daily arsenic exposures for adults and children 4–30 months of age and cancer risks among adult consumers.

**Results:**

Turkey meat from conventional producers not prohibiting nitarsone use showed increased mean levels of iAs (0.64 μg/kg) and MA (5.27 μg/kg) compared with antibiotic-free and organic meat (0.39 μg/kg and 1.54 μg/kg, respectively) and meat from conventional producers prohibiting nitarsone use (0.33 μg/kg and 0.28 μg/kg, respectively). Samples with measurable nitarsone had the highest mean iAs and MA (0.92 μg/kg and 10.96 μg/kg, respectively). Nitarsone was higher in October samples than in March samples, possibly resulting from increased summer use. Based on mean iAs concentrations in samples from conventional producers with no known policy versus policies prohibiting nitarsone, estimated lifetime daily consumption by an 80-kg adult, and a recently proposed cancer slope factor, we estimated that use of nitarsone by all turkey producers would result in 3.1 additional cases of bladder or lung cancer per 1,000,000 consumers.

**Conclusions:**

Nitarsone use can expose turkey consumers to iAs and MA. The results of our study support the U.S. Food and Drug Administration’s removal of nitarsone from the U.S. market and further support its removal from the global marketplace.

**Citation:**

Nachman KE, Love DC, Baron PA, Nigra AE, Murko M, Raber G, Francesconi KA, Navas-Acien A. 2017. Nitarsone, inorganic arsenic, and other arsenic species in turkey meat: exposure and risk assessment based on a 2014 U.S. market basket sample. Environ Health Perspect 125:363–369; http://dx.doi.org/10.1289/EHP225

## Introduction

Arsenic-based drugs have been used in the production of chickens, turkeys, and swine in the United States since the 1940s (Silbergeld and Nachman 2008). The approvals for three of these drugs, roxarsone, arsanilic acid, and carbarsone, were withdrawn by the U.S. Food and Drug Administration (FDA) on 30 September 2013, rendering their domestic sale illegal (FDA 2013). The approval of a fourth drug, nitarsone [(4-nitrophenyl)arsonic acid, C_6_H_6_AsNO_5_], used in chickens and turkeys, was withdrawn by the FDA in December of 2015, terminating the domestic sale of the drug.

Evidence shows that the use of arsenic-based drugs in food animal production results in human dietary exposures to arsenic, including inorganic arsenic (iAs) (Lasky et al. 2004; Wallinga 2006; Nachman et al. 2013; Liu et al. 2015, 2016), and in the environmental distribution of arsenic in manure (Jackson and Bertsch 2001; Bednar et al. 2003; Garbarino et al. 2003; Jackson et al. 2003; Rutherford et al. 2003; Nachman et al. 2005, 2008). Previous research on arsenic-based drugs has primarily considered roxarsone, a drug used in ≤ 90% of domestic chicken production before its removal from the U.S. market (Nachman et al. 2012). In a U.S.-based market-basket study, we found significant increases in iAs concentrations in chicken meat from animals likely raised with roxarsone compared with meat from organic and antibiotic-free chickens not fed roxarsone (Nachman et al. 2013).

**Figure 1:**
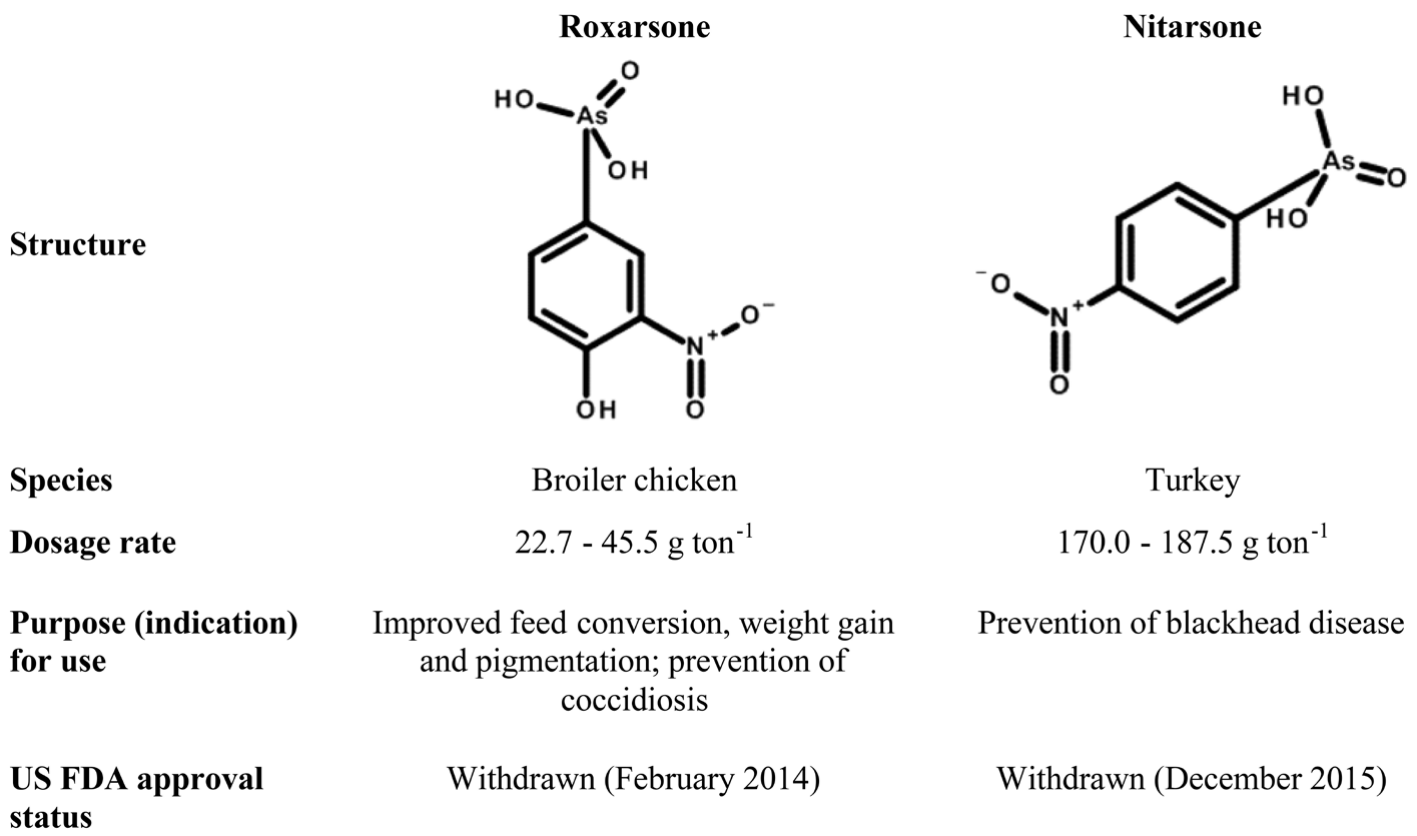
Comparison of two arsenical poultry drugs, roxarsone and nitarsone. Dosage rate and indication information are from the Food and Drug Administration’s Animal Drugs @ FDA website [U.S. Food and Drug Administration (FDA)].

Little is known, however, about the potential arsenic exposure resulting from nitarsone use in turkey production. Its clinical indication is for the prevention of blackhead disease in poultry, which is caused by the protozoan species *Histomonas meleagridis* (McDougald 2005). To our knowledge, no study has evaluated the distribution of arsenic species in meat from nitarsone-treated turkeys. Given the similarity of roxarsone and nitarsone (Figure 1), nitarsone use could result in similar dietary exposures to arsenic for turkey consumers.

The level of nitarsone dosage during its preventive use and the lifespan of turkeys raised for meat consumption suggest that arsenic species could accumulate in commonly consumed tissues such as muscle, fat, and skin. Although information regarding pharmaceutical use in animal production is not made public, statements from industry trade groups suggest that nitarsone was widely used in U.S. turkey production before its withdrawal from the market in 2015, mostly during the first few weeks of the birds’ lives and more heavily during summer months, in turkeys to be consumed during the fall and winter (Aubrey 2013; Strom 2013).

The purpose of this study was to examine the potential impact of nitarsone use on arsenic species exposure among turkey consumers. Specifically, we studied retail turkey products to characterize the occurrence of arsenic species in meat. Given industry statements about the seasonality of nitarsone usage in turkey production, we examined differences in the occurrence of arsenic species in meat from turkey products purchased in two different seasons. Using turkey consumption data from the National Health and Nutrition Examination Survey (NHANES), we estimated the lifetime average daily exposure to these arsenic species among turkey consumers and estimated cancer risks associated with ingestion of iAs attributable to arsenical drug use in animal production.

## Methods

### Sample Collection and Preparation

A total of 184 turkey samples were included in our study, including products from 14 producers representing 64% of the 2014 U.S. turkey market (National Turkey Federation 2016). Turkeys reach market weight and are processed at 4–5 months of age. Given this information, we examined 128 samples of raw turkey meat purchased in March (to represent turkeys whose lifespan primarily did not overlap with summer months when nitarsone use may have been less likely or reduced) and 56 raw turkey samples purchased in October (to represent turkeys whose lifespan largely overlapped with the summer months when nitarsone use was more likely).

Samples purchased in March 2014 were purchased from retail grocers in three geographically diverse U.S. metropolitan areas (Baltimore, MD; Denver, CO; and Los Angeles, CA), selected to cover the East Coast, the West Coast, and the Midwest. We visited 12 unique stores (9 supermarket chains) in Los Angeles, 10 unique stores (6 supermarket chains) in Denver, and 12 unique stores (10 supermarket chains) in Baltimore, for a total of 36 unique stores representing 23 supermarket chains. All samples collected in October 2014 (*n* = 56) were purchased in Baltimore, Maryland; 10 additional samples were purchased in October (compared with Baltimore samples from March, *n* = 46) to increase the study sample size for that month. There was slight variation in grocery chains and stores visited in March and October, but overall, there was considerable overlap in purchase sites.

All samples were taken from packages of raw turkey meat (ground or whole cuts). Within each store, we purchased two packages of a single type (ground or whole cuts) of turkey for each brand available (multiple brands were purchased when available within a store), including store-branded products. We also purchased conventionally produced products and U.S. Department of Agriculture (USDA) Organic and antibiotic-free–labeled turkey products of each type and brand, when possible. If only one package was available for a type and brand of turkey, then one single package was purchased. For example, from a given store in one of the study cities, based on available products, we purchased two packages of conventional ground turkey and two packages of conventional whole cut turkey from Producer B and two packages of conventional ground turkey from Producer A. In this particular store, neither antibiotic-free nor USDA Organic turkey products were available.

The mean [standard deviation (SD)] meat weight in the purchased package was 0.52 (0.15) kg for ground meat and 0.69 (0.28) kg for whole cuts. From each package, 60- to 70-g aliquots of meat were collected and processed as described below for arsenic analyses. All whole pieces of meat examined were exclusively breast tissue. Ground meat, however, was often a mixture of breast and other unspecified muscle tissue. We did not purchase cooked products, deli meats, or products such as hot dogs, sausages, or frozen meals containing turkey because mixed ingredients in the packages could interfere with sample analysis. For example, some packages of turkey deli meat contain rice starch or carrageenan (an edible extract from algae), which are used for gelling and thickening and could have been a source of added arsenic.

Samples were prepared for freeze-drying as previously described (Nachman et al. 2013). Briefly, a roughly 60- to 70-g subsample of raw meat was removed from each sample package. The subsample was > 10% of the package weight (the mean package weight was 560 g), which we assumed to be representative of the entire package. The subsamples were individually homogenized in a food processor with the addition of 75 mL MilliQ water (Millipore Corporation) to aid blending. Blended samples were weighed and stored in sealable bags at 20°C. Between samples, the food processor and all laboratory equipment were cleaned with hot water, soaked for 30 min in a 10% nitric acid bath, and rinsed with MilliQ water. Frozen sample homogenates were shipped on dry ice to Oregon State University for freeze-drying (SP Scientific freeze dryer). Sample-specific water loss factors were recorded. Freeze-dried samples were stored as a crumbled powder in 50-mL polypropylene tubes at 25°C and were shipped from Johns Hopkins University to the Institute of Chemistry-Analytical Chemistry, University of Graz, Austria, for arsenic analyses.

### Determination of Total Arsenic and Arsenic Species

Detailed descriptions of laboratory reagents, standards, and reference materials; instrumentation; extraction of arsenic species; and HPLC-ICPMS analyses are provided in “Detailed Laboratory Methods Section” in the Supplemental Material. In brief, the total arsenic contents of the freeze-dried turkey samples were determined using inductively coupled plasma mass spectrometry (ICPMS–Agilent 7900 ICPMS from Agilent Technologies) following microwave-assisted acid mineralization in an UltraCLAVE III microwave system (MLS GmbH). Arsenic standards in the range 0.01–20 μg As/L were used for external calibration; germanium (20 μg/L in final solution) was added to standards and samples to normalize matrix effects. Because there is currently no turkey or chicken meat reference material certified for total arsenic content, we used the standard reference material ERM-BC211 rice (European Commission 2012) for quality control; this material has a certified arsenic content of 260 ± 13 μg As/kg; we obtained 265 ± 18 μg As/kg (*n* = 39) over the course of the study. The turkey samples were analyzed in duplicate for their total arsenic content; when duplicate values differed by > 10%, which occurred with only 4 of the 184 samples, the sample was re-analyzed. Taken together, 432 total arsenic measurements (including duplicate measurements for the 184 samples, 4 additional measurements for outliers, and duplicate measurements for 30 blinded duplicate quality control samples) were made.

Arsenic species were determined in duplicate analysis in alkaline aqueous extracts of the freeze-dried turkey samples by using anion-exchange high performance liquid chromatography (HPLC) coupled to an ICPMS which served as an arsenic-selective detector. When the values for arsenic species in the duplicates differed by > 20%, the sample was re-analyzed. Separation of dimethylarsinate (DMA), methylarsonate (MA), iAs, and nitarsone was achieved by gradient elution using ammonium carbonate buffer with a Dionex AS15A anion exchange column. For quality control, we used the standard reference material ERM-BC211 (rice, certified contents of DMA 119 ± 13 μg As/kg and iAs 124 ± 11 μg As/kg); we obtained 116 ± 4 μg As/kg for DMA and 99 ± 4 μg As/kg for iAs (*n* = 9). Detection limits, on a dry weight basis, were 1, 1–2, 1, 1, and 1 μg/kg for total arsenic, iAs, MA, DMA, and nitarsone, respectively.

Sample-specific concentration estimates were derived by taking the average of the replicate measurements (2–3 replicates for total arsenic and each arsenic species). For total arsenic, iAs, MA, and DMA measurements below the detection limit, we imputed the value of the detection limit divided by the square root of 2. For nitarsone, samples below the detection limit were given the value of 0. For each arsenic species, wet weight sample concentrations were calculated for each sample by multiplying its dry weight concentration by the sample-specific water loss dilution factor.

Samples were analyzed in a random order, and the lab was blinded to sample identity. Thirty blinded duplicate samples were analyzed separately to evaluate the performance of the method but were not included as additional samples in the main analysis.

### Other Variables

We categorized samples into two groups based on their package labels to compare USDA Organic or antibiotic-free to conventionally produced samples. It is critical to note that although the USDA Organic certification program explicitly prohibits the use of arsenic-based pharmaceuticals in animal production (USDA 2016b), the label “no antibiotics added” only restricts the use of antibiotics and does not specifically prohibit arsenical antimicrobials (USDA 2015). In addition, we classified each of the 117 conventional samples (not labeled as USDA Organic or as antibiotic-free) as being from a conventional producer with a stated policy against nitarsone use (one producer, 30 samples) or from a conventional producer with no known policy against nitarsone use (eight producers, 87 samples) based on information from company websites or from responses to emails or phone calls requesting this information. After laboratory analyses, all samples were further categorized based on the presence of measurable nitarsone above the detection limit.

### Statistical Analyses

Statistical analyses were performed with Stata 14 (StataCorp LLC). Mean arsenic species concentrations and 95% confidence intervals (CIs) were calculated to evaluate differences among categories of turkey samples (by package label, by nitarsone policy, by positive nitarsone detection, and by season of purchase). The seasonal analysis was also restricted to Baltimore-only samples in a sensitivity analysis. Pearson’s coefficients were used to assess correlations between concentrations of total arsenic and arsenic species including nitarsone. Statistical significance was two-tailed and was set at α = 0.05.

### Estimation of Population Turkey Consumption Rates and Body Weights

***Intake rate calculation.*** Population intake rates for turkey meat were derived from the dietary recall component of the 2003–2010 cycles of the National Health and Nutritional Examination Survey (NHANES) [National Center for Health Statistics (NCHS) 2015] using the “survey” package in R (version 3.30; R Project for Statistical Computing) to account for NHANES’s complex survey design and sampling weights (Lumley 2014); detailed survey and dietary recall methods are available through the National Center for Health Statistics (NCHS 2014). Each reported food item is linked to a systematic, 8-digit USDA food code and is recorded in grams. Because USDA food code items often contain multiple food components (e.g., “turkey sandwich”), we linked participant information with the U.S. Environmental Protection Agency’s (EPA’s) Food Commodity Index Database (FCID), developed by the Office of Pesticide Programs (OPP) for use in pesticide risk analysis (Joint Institute for Food Safety and Applied Nutrition 2003, 2008). The FCID converts the total weight of each USDA food code item into the weight attributable to each commodity (e.g., “Turkey, meat”). Each commodity weight is summed across all USDA food items and expressed in grams per kilogram body weight per day. Because the FCID database was updated in 2005, we merged the original database for 2003–2004 participants with the updated database for 2005–2010 participants. We derived the consumer-only intake rate from any participant reporting consumption of a USDA food code item containing the FCID commodity code 5000382000, “Turkey, meat.” The commodities “Turkey, skin,” “Turkey, fat,” “Turkey, liver,” and “Turkey, meat byproducts,” do not contribute to our intake rate calculations because “Turkey, meat” best corresponds to the turkey breast meat analyzed in our study. This intake rate represents the average consumption of turkey meat, in grams per kilogram body weight per day, of all individuals who reported any turkey consumption on either day of dietary recall (24.1%).

The consumer-only intake rate for turkey in baby food was derived similarly among participants age 4–30 months using the FCID commodity code 5000382001, “Turkey, meat-babyfood.” Because the small number of participants consuming the commodity resulted in strata with one primary sampling unit, we conservatively centered single-primary sampling unit strata at the grand mean (Lumley 2014).

Among the approximately 24% of the U.S. population that consumes “Turkey, meat,” intake rates were estimated at 0.49 (95% CI: 0.47, 0.51) g/kg BW/day; intake rates of “Turkey, meat-babyfood” were estimated at 1.86 (95% CI: 1.50, 2.23) g/kg BW/day for children 4–30 months of age.

***Body weight estimation.*** The adult body weight distribution for the risk assessment of “Turkey, meat” was derived from all participants ≥ 18 years old, and the body weight distribution for “Turkey, meat-babyfood” was derived from all participants 4–30 months old.

### Exposure and Risk Analyses

To estimate the arsenic species exposure burden attributable to turkey consumption in adults, we estimated the lifetime average daily doses (LADD) for each arsenic species for each of the categories of turkey products (by package label, by nitarsone use policy, and by positive nitarsone detection) using the formula:

LADD = ([As] × IR)/BW, [1]

where LADD is the lifetime average daily dose (in milligrams per kilogram body weight per day), [As] is the mean arsenic species concentration (in milligrams per kilogram) in the specific category of turkey product, IR is the per capita turkey intake rate, and BW is body weight (80 kg BW) (U.S. EPA 2011).

We also estimated average daily doses of arsenic species for children between the ages of 4 and 30 months who consume baby food made from turkey using the formula:

ADD = ([As] × IR)/BW, [2]

where ADD is the average daily dose (in milligrams per kilogram body weight per day), [As] is the mean arsenic species concentration (in milligrams per kilogram), IR is the turkey intake rate (0.019 kg/day), and BW is body weight (11.01 kg BW).

For iAs, we also estimated the difference in iAs intake for consumers of turkey produced with nitarsone compared with consumers of turkey produced without nitarsone by subtracting the mean iAs concentration for the antibiotic-free or USDA-certified Organic group from the mean iAs concentration for the “conventional with no known arsenical policy” group. The resulting value is an estimate of the added iAs in the meat from the use of nitarsone. The calculation of the LADD and the risk using this value reflects an estimation of the added iAs exposure and excess lifetime cancer risk attributable to the decision to use nitarsone in turkey production. We then estimated cancer risk in turkey consumers by multiplying this LADD by the U.S. EPA Integrated Risk Information System (IRIS) cancer slope factor (q*) for iAs:

Risk = LADD_iAs_ × q*. [3]

The U.S. EPA IRIS toxicological review of inorganic arsenic is currently under reassessment and review by the National Research Council; consequently, we used a value of 25.7/[mg/kg BW/day] proposed in a draft version of the toxicological review in 2010, reflecting the U.S. EPA’s most recently published analysis of the epidemiologic literature and corresponding to lung and bladder cancers (U.S. EPA 2010). Toxicity metrics were unavailable for other species. Population cancer burdens were calculated by multiplying the estimated cancer risk by the fraction (24%) of the 2015 U.S. population consuming turkey (76,821,806).

We conducted sensitivity analyses to examine how the relative market share of each turkey producer would affect the estimated population cancer burden attributable to turkey consumption in the United States if nitarsone had not been withdrawn from the market. Using the most recent (2014) producer-specific data (see Table S1) from the National Turkey Federation (2016), a trade association for the U.S. turkey industry, we calculated the relative market share for each producer by dividing its 2014 production quantity by the production quantity summed across all producers. We then multiplied this market share by the mean iAs value (from our sampling data) for each producer [for turkey producers for whom we did not test samples, we imputed the average iAs value from the study (0.50 μg/kg)] and then summed those values to create a weighted mean iAs concentration for turkey products in the United States (0.72 μg/kg). We then used this weighted mean iAs in the previously described models to estimate LADD, risk, and annual population cancer burden.

A second approach was considered to allow for comparisons between producers who likely use nitarsone and those who do not. Considering only turkey producers for whom we have samples (representing 64% of the U.S. turkey market), we used the 2014 producer-specific data from the National Turkey Federation to calculate the relative market share for each producer by dividing its 2014 production quantity by the production quantity summed across the producers included in our study. We then categorized producers into a nitarsone-positive group (including producers with at least one sample with measurable nitarsone) and a nitarsone-negative group (including producers with no measurable nitarsone in any sample). Then, weighted mean iAs concentrations were calculated for the nitarsone-positive (0.74 μg/kg) and nitarsone-negative (0.11 μg/kg) groups separately. The difference in those weighted means (0.63 μg/kg) was considered the iAs attributable to nitarsone use. This value was then used in the previously described models to estimate LADD, risk, and annual population cancer burden.

## Results

### Arsenic Species in Turkey Meat

The mean total arsenic concentration in turkey meat was 11.2 (95% CI: 7.2, 15.1) μg/kg (Table 1). Mean iAs, MA, DMA, and nitarsone concentrations were 0.5 (95% CI: 0.4, 0.6), 3.1 (95% CI: 2.0, 4.2), 2.4 (95% CI: 2.1, 2.6), and 0.3 (95% CI: 0.1, 0.4) μg/kg, respectively. Nitarsone was detected in 17% of samples.

**Table 1:** Mean (95% confidence interval) of concentrations of total arsenic and arsenic species in turkey meat by sample characteristics.

Turkey sample classification	*n*	Total arsenic mean (95% CI) (μg As/kg)	Arsenic species mean (95% CI)				
			iAs (μg As/kg)	MA (μg As/kg)	DMA (μg As/kg)	Nitarsone (% positive)	Nitarsone (μg As/kg)
All	184	11.18 (7.24, 15.11)	0.50 (0.41, 0.59)	3.10 (1.99, 4.21)	2.37 (2.10, 2.64)	17	0.27 (0.10, 0.43)
Package label
Conventional	117	14.53 (8.42, 20.64)*	0.56 (0.43, 0.70)	3.99 (2.27, 5.72)*	2.49 (2.12, 2.87)	21	0.39 (0.13, 0.65)
Antibiotic-free or organic	67	5.32 (4.33, 6.32)	0.39 (0.35, 0.43)	1.54 (1.21, 1.87)	2.16 (1.80, 2.52)	10	0.054 (0.014, 0.094)
Producer arsenical policy
Conventional with no known policy	87	19.20 (11.18, 27.21)**	0.64 (0.46, 0.82)*	5.27 (3.01, 7.54)*	3.09 (2.65, 3.53)***	28	0.53 (0.18, 0.87)
Conventional with prohibitory policy	30	0.98 (0.82, 1.15)	0.33 (0.29, 0.37)	0.28 (0.24, 0.32)	0.76 (0.65, 0.86)	0	NA
Nitarsone detection
Negative	153	5.15 (4.40, 5.91)***	0.42 (0.36, 0.47)***	1.51 (1.23, 1.78)***	1.94 (1.74, 2.14)***	0	NA
Positive	31	40.89 (19.97, 61.81)	0.92 (0.51, 1.33)	10.96 (5.01, 16.90)	4.52 (3.51, 5.52)	100	1.59 (0.72, 2.47)
Month of purchase
March (Baltimore only)	46	7.58 (4.95, 10.21)	0.40 (0.22, 0.58)**	2.45 (1.47, 3.45)	2.08 (1.69, 2.47)	20	0.11 (0.04, 0.19)
October (Baltimore only)	56	18.77 (6.34, 31.20)	0.79 (0.57, 1.02)	5.33 (1.86, 8.80)	2.47 (1.80, 3.15)	20	0.65 (0.12, 1.19)
Metropolitan area
Baltimore, Maryland	102	13.73 (6.81, 20.64)	0.62 (0.47, 0.78)	4.04 (2.08, 5.99)	2.30 (1.89, 2.70)	20	0.41 (0.12, 0.70)
Denver, Colorado	40	5.30 (4.05, 6.54)	0.31 (0.27, 0.36)	1.46 (0.96, 1.96)	2.42 (1.94, 2.91)	10	0.053 (0, 0.11)
Los Angeles, California	42	10.59 (6.81, 14.36)	0.40 (0.34, 0.46)	2.39 (1.45, 3.33)	2.51 (1.99, 3.02)	17	0.13 (0.027, 0.23)
Processor
Producer A	6	12.68 (4.02, 21.34)	1.71 (0.41, 3.01)	1.35 (–0.06, 2.77)	1.80 (1.08, 2.52)	17	0.30 (0.48, 1.08)
Producer B	34	31.56 (11.64, 51.47)	0.77 (0.39, 1.16)	9.17 (3.57, 14.77)	4.13 (3.17, 5.10)	44	1.09 (0.24, 1.95)
Producer C	14	3.71 (0.98, 6.44)	0.36 (0.28, 0.44)	0.90 (0.32, 1.48)	1.59 (0.40, 2.79)	21	0.13 (0, 0.27)
Producer D	14	6.70 (5.61, 7.80)	0.61 (0.47, 0.74)	1.47 (0.98, 1.95)	2.88 (2.23, 3.53)	0	NA
Producer E	30	0.98 (0.82, 1.15)	0.33 (0.29, 0.37)	0.28 (0.24, 0.32)	0.76 (0.65, 0.86)	0	NA
Producer F	27	5.24 (4.13, 6.35)	0.28 (0.24, 0.32)	1.34 (0.79, 1.90)	1.95 (1.56, 2.33)	7	0.068 (0, 0.16)
Producer G	20	4.54 (3.24, 5.84)	0.39 (0.34, 0.45)	1.72 (1.22, 2.22)	1.80 (1.36, 2.25)	10	0.053 (0, 0.13)
Producer H	8	32.87 (25.42, 40.32)	0.47 (0.36, 0.57)	7.86 (6.05, 9.67)	4.12 (3.05, 5.19)	75	0.63 (0.20, 1.06)
Producer I	6	8.14 (5.37, 10.91)	0.43 (0.32, 0.53)	3.59 (2.41, 4.78)	1.86 (1.43, 2.28)	0	NA
Producer J	6	6.29 (4.87, 7.70)	0.33 (0.20, 0.46)	1.60 (0.58, 2.62)	2.25 (1.93, 2.57)	0	NA
Other producers	19	7.91 (5.72, 10.09)	0.45 (0.36, 0.54)	2.34 (1.48, 3.20)	2.82 (2.38, 3.26)	10	0.04 (0, 0.10)
Notes: As, arsenic; CI, confidence interval; DMA, dimethylarsinate; iAs, inorganic arsenic; MA, methylarsonate; NA, not applicable, all samples below the detection limit. Detection limits were reported in dry weight as 1, 1–2, 1, 1, and 1 μg As/kg for total arsenic, iAs, MA, DMA and nitarsone, respectively. Sample-specific concentration estimates were derived by taking the average of the replicate measurements from the same package (2–3 replicates for total arsenic and each arsenic species). For total arsenic, iAs, MA and DMA measurements below the detection limit, we imputed the value of the detection limit divided by the square root of 2. For nitarsone, samples below the detection limit were given the value of 0. *p < 0.05; **p < 0.01; ***p < 0.001.							

By package label, conventionally produced samples (i.e., those coming from turkeys permitted to receive nitarsone) had higher total arsenic (*p* = 0.026), iAs (*p* = 0.041), and MA (*p* = 0.036) concentrations than the combined USDA Organic and antibiotic-free labeled samples. Measurable nitarsone was observed in 21% of conventional samples, compared with 10% nitarsone in the combined antibiotic-free and USDA Organic samples. Within the conventional group, further differences were observed when samples were grouped by whether producers had a known policy or no known policy prohibiting arsenic use. Samples from conventional producers with no known policy (*n* = 87 samples) had significantly higher mean concentrations for all arsenic species than samples from conventional producers with prohibitory policies (*n* = 30 samples, *p* < 0.05 for all comparisons). Nitarsone was not found in samples from conventional producers with prohibitory policies but was detected in 28% of samples from producers without known policies.

The greatest differences in arsenic species concentrations were observed between samples with and without measurable nitarsone (*n* = 31 and 153 samples, respectively, *p* < 0.01 for all comparisons) (Table 1).The largest difference was for MA, with a mean of 11.0 (95% CI: 5.0, 16.9) μg/kg for samples with detectable nitarsone compared with 1.5 (95% CI: 1.2, 1.8) μg/kg for samples without detectable nitarsone (*p* < 0.001).

Seasonal comparisons were examined using only Baltimore samples. A significant difference in iAs (in micrograms per kilogram) was observed (*p* = 0.009) between samples purchased in March [0.40 (95% CI: 0.22, 0.60)] and in October [0.79 (95% CI: 0.57, 1.02)]. Nitarsone concentrations were 0.65 (95% CI: 0.12, 1.19) μg/kg for samples purchased in October and 0.11 (95% CI: 0.04, 0.19) μg/kg for samples purchased in March (*p* = 0.07). Differences were not observed for the other species (*p*-values were all > 0.1), although the mean total arsenic and MA concentrations were higher for samples purchased in October.

Nitarsone residues were measured in at least one sample from 7 of the 19 producers included in the study (producers with three or fewer samples were grouped into an “other” category); among those, the frequency of nitarsone detection varied considerably, from 7% to 100%. The producer with a rate of 100% nitarsone measurement was part of the “other” category and accounted for two samples; none of the remaining producers (*n* = 8) in the “other” category had measurable nitarsone. Among producers with measurable nitarsone, mean values ranged from 0.05 to 1.09 μg/kg.

We estimated Pearson correlation coefficients between arsenic species (see Table S2). Moderate to strong correlations were observed for all measured arsenic species, with the weakest correlation being between iAs and DMA (0.54).

### Exposure and Risk Analyses

Estimates for LADDs of arsenic species by sample characteristics are presented in Table 2. Consuming turkey raised under different management regimens changed the estimated daily exposures to arsenic species and total arsenic. For example, for adults and children, individual and combined arsenic species exposures were more than two times higher from turkeys raised by conventional producers with no nitarsone policies than from turkeys raised by conventional producers with policies banning its use. For the conventional, conventional with no known arsenical policy, and positive nitarsone detection sample categories, MA exposure accounted for the largest contribution for a single species; for the other categories, DMA contributed the most to exposure.

**Table 2:** Estimates of lifetime average daily dose of arsenic species in adults and average daily dose in children (in micrograms per kilogram BW per day) resulting from consumption of turkey, based on sample characteristics.

Sample characteristic	iAs	MA	DMA	Nitarsone
Adult
Antibiotic-free or USDA-certified Organic	0.00019	0.00075	0.00106	0.00003
Conventional, all	0.00028	0.00196	0.00122	0.00019
Conventional with prohibitory policy	0.00016	0.00014	0.00037	NA
Conventional, no known arsenical policy	0.00031	0.00260	0.00146	0.00026
No nitarsone detection	0.00020	0.00074	0.00095	NA
Positive nitarsone detection	0.00045	0.00537	0.00222	0.00078
Child (4–30 months of age)
Antibiotic-free or USDA-certified Organic	0.00073	0.00280	0.00390	0.00010
Conventional, all	0.00105	0.00720	0.00450	0.00073
Conventional with prohibitory policy	0.00061	0.00051	0.00150	NA
Conventional, no known arsenical policy	0.00119	0.00950	0.00560	0.00093
No nitarsone detection	0.00077	0.00270	0.00350	NA
Positive nitarsone detection	0.00171	0.02039	0.00820	0.00300
Notes: As, arsenic; CI, confidence interval; DMA, dimethylarsinate; iAs, inorganic arsenic; MA, methylarsonate; NA, not applicable, all samples below the detection limit; USDA, U.S. Department of Agriculture. Arsenic species mean concentration estimates from Table 1 were used to calculate daily doses. Turkey meat intake rates of 0.49 and 1.86 g/kg BW/day, calculated from National Health and Nutrition Examination Survey (NHANES) dietary intake data, were used for adults and for children 4–30 months of age, respectively, in estimation of daily doses. Body weights of 80 and 11 kg (estimated from NHANES data) were used for adults and for children 4–30 months of age, respectively.				

By group, iAs exposures were higher among samples from conventional producers without policies prohibiting nitarsone use and samples with measurable nitarsone and lower among samples from the combined antibiotic-free and USDA Organic group and conventional producers where use was prohibited by company policy. Similar patterns were seen for average daily doses for children (Table 2). One notable difference, however, is that considering their differing intake rates and body weights, exposures among children 4–30 months of age were almost four times higher than those for adults (e.g., when considering turkey with a positive nitarsone detection, average daily iAs doses for children 4–30 months of age were 1.7 × 10^–6^ mg/kg BW/day, compared with 4.5 × 10^–7^ mg/kg BW/day for adults).

Compared with consumers of antibiotic-free and USDA Organic turkey, we estimated that an average 80-kg person consuming 0.039 kg turkey per day from conventional producers without policies prohibiting nitarsone use (mean iAs concentration of 0.00064 mg/kg) would ingest an additional 0.01 μg iAs per day, resulting in a LADD of 1.22 × 10^–7^ mg/kg BW/day. Based on the U.S. EPA’s proposed cancer slope factor for iAs of 25.7/(mg/kg BW/day) (U.S. EPA 2010), this lifetime average daily exposure would result in approximately 3.1 additional cases of bladder or lung cancer per 1,000,000 persons. Our estimates for the 2015 U.S. population (U.S. Census Bureau 2014), 24% of whom are estimated to be turkey consumers, suggest that if turkey industry use of nitarsone had not been discontinued, 241 additional cases of cancer might have occurred in the United States over 70 years (3.4 cancers per year in the U.S. population as a whole). This scenario represents the estimated increase in cancer cases if nitarsone was used in all domestically produced turkey in the United States and people were exposed throughout their lives compared with the numbers of cases expected in the absence of any nitarsone use by turkey producers in the United States.

We conducted additional sensitivity analyses to consider the influence of the market share of turkey producers on cancer burden estimates had nitarsone not been withdrawn. Using 2014 producer-specific data from the National Turkey Federation (2016), we estimated the market share–adjusted population cancer burden from iAs resulting from consumption of turkey. Under this scenario, which employed market share–weighted iAs producer-specific concentration estimates (and the mean iAs value from our study imputed for producers we did not sample), we estimated an iAs LADD of 3.53 × 10^–7^ mg/kg BW/day, a risk of 9.07 × 10^–6^, and an annual population cancer burden of 10 cases per year attributable to turkey consumption in the United States (including contributions from both nitarsone users and nonusers). To refine this estimate of burden, we estimated the market share–adjusted population cancer burden from iAs attributable to nitarsone use by separating producers into nitarsone use and nonuse categories based upon our data (producers not sampled in our study were excluded from this analysis). Under this scenario, we estimated a LADD of 3.09 × 10^–7^ mg/kg BW/day, a risk of 7.93 × 10^–6^, and an annual population cancer burden of 9 cases per year. Burden estimates under these two modeled scenarios were higher than the original burden estimation, which did not consider producer market share. The reason for this difference is the uneven market share across producers; for example, the producer with the largest market share was a nitarsone user with the highest mean iAs concentration.

Because cancer slope factors are not available for other arsenic species, the contributions of MA and DMA to lung and bladder cancer risks cannot be estimated despite evidence supporting their potential carcinogenicity [International Agency for Research on Cancer (IARC) 2004].

## Discussion

Before their withdrawals in 2014 and 2015 (FDA 2015), arsenic-based drugs had been used in the United States since the 1940s (Silbergeld and Nachman 2008). Our results support the hypothesis that nitarsone use increases inorganic and methylated arsenic species concentrations in turkey meat, resulting in a source of arsenic exposure for consumers of treated birds. We found that estimated arsenic exposure differed according to the month of purchase, which is consistent with an effect of seasonal patterns of nitarsone on residues. This finding is consistent with an analysis of NHANES data collected before withdrawal of arsenical medications for use in poultry suggesting that seasonal variation in turkey consumption contributed to seasonal variation in urinary arsenic concentrations in the United States (Nigra et al. 2016). In addition, we found that MA concentrations were significantly elevated in samples where nitarsone was measured above the detection limit (compared with samples where nitarsone was not found above the detection limit). Epidemiologic data describing the biological significance of MA are limited. Experimental research suggests, however, that trivalent forms of these species, which are products of methylation processes in human metabolism of arsenic (Aposhian et al. 2004), are genotoxic (Styblo et al. 2000; Mass et al. 2001; Kligerman et al. 2003); in mice, maternal exposures during gestation have been shown to elicit tumors at multiple sites (uterine, ovarian, and adrenal tumors in females, and hepatocellular, adrenal, and lung in males) in offspring (Tokar et al. 2012). Because epidemiologic investigations have focused on drinking water (where iAs dominates), little is known about the health significance of oral exposure to methylated species, though concerns have been raised about the potential for their carcinogenicity in humans by IARC and others (IARC 2004; deCastro et al. 2014).

Our results come soon after the FDA’s withdrawal of the marketing approval of nitarsone in the United States (FDA 2015). The basis for the FDA’s commitment to withdraw nitarsone’s approval was the body of research surrounding roxarsone (FDA 2011; Conklin et al. 2012; Nachman et al. 2013; Peng et al. 2014; Liu et al. 2015) because no data specific to nitarsone have been available. Our analyses provide support for the FDA’s precautionary action. In addition to mitigating dietary arsenic exposure, ending domestic nitarsone sales will eliminate the introduction of arsenic into the turkey production manure stream, removing a significant contribution of arsenic from the U.S. food production cycle.

The FDA action against arsenical drugs in the United States does not affect their use in other countries, nor does it prevent U.S.-based pharmaceutical companies from selling these drugs in other countries. For example, arsenicals are approved for use in China and can be found in commercial feed for poultry and swine and in animal manure in China (Yao et al. 2013; L Huang et al. 2014; K Huang et al. 2015). The USDA is close to approving exports of cooked poultry products from China for the U.S. market; these products would be raised, slaughtered, and processed in China (USDA 2016a). If these imports occur, U.S. consumers may face arsenical exposures from poultry meat. Independent of U.S. concerns, Chinese consumers also face health risks from consuming poultry and swine fed arsenical drugs. To avoid unnecessary exposures to arsenic species, we encourage the global withdrawal of arsenical drugs through revision to the Food and Agriculture Organization of the United Nations/World Health Organization (FAO/WHO) Codex Alimentarius.

Our assessment did not specifically address noncancer health concerns linked to chronic iAs exposure [National Research Council (NRC) 2014], and little is known about hazards related to routine exposure to other arsenic species. In addition, our earlier work on roxarsone suggested that cooking changed the species profile, leaving methylated species intact but significantly decreasing the parent compound in favor of the production of iAs (Nachman et al. 2013). It is unknown if cooking also affects nitarsone, but if cooking has an effect similar to its apparent effect on roxarsone, we may have underestimated potential iAs exposures to consumers of nitarsone-treated turkey meat because our estimates were based on measured concentrations in raw turkey.

We were unable to account for other potential sources of arsenic that could be related to differences in the impact of nitarsone use on measured arsenic species, including arsenic in the drinking water used by turkeys or nonarsenical drug–related sources of arsenic in the feed used for turkeys.

Future work (much of which is dependent on the development of quantitative toxicity metrics for noncancer health outcomes for iAs and cancer potency factors for other arsenic species) is needed to contextualize other aspects of the current and historic health burden imposed on turkey consumers by the use of nitarsone.

## Conclusion

Our study provides evidence that the use of nitarsone in turkey production can contribute to exposure to iAs and methylated arsenic species among turkey consumers. Our findings support the FDA’s precautionary decision to withdraw marketing approval for nitarsone in 2015, and they support similar actions by other governments and international agencies to protect public health in all populations.

## Supplemental Material

(159 KB) PDF
