# Supplementary material for: Nitarsone, Inorganic Arsenic, and Other Arsenic Species in Turkey Meat: Exposure and Risk Assessment Based on a 2014 U.S. Market Basket Sample
Source: Environ Health Perspect. 2016 Oct 13;125(3):363–9. doi: 10.1289/EHP225 (PMC5332177; doi:10.1289/EHP225)
Supplement: (159 KB) PDF [file EHP225.s001.acco.pdf]

Environ Health Perspect
DOI: 10.1289/EHP225

Note to readers with disabilities: *EHP* strives to ensure that all journal content is accessible to all readers. However, some figures and Supplemental Material published in *EHP* articles may not conform to 508 standards due to the complexity of the information being presented. If you need assistance accessing journal content, please contact ehp508@niehs.nih.gov. Our staff will work with you to assess and meet your accessibility needs within 3 working days.

Supplemental Material

Nitarsone, Inorganic Arsenic, and Other Arsenic Species in Turkey Meat: Exposure and Risk Assessment Based on a 2014 U.S. Market Basket Sample

Keeve E. Nachman, David C. Love, Patrick A. Baron, Anne E. Nigra, Manuela Murko, Georg Raber, Kevin A. Francesconi, and Ana Navas-Acien

Table of Contents

DETAILED LABORATORY METHODS SECTION **2**
Reagents, standards and reference materials *2*
Instrumentation *2*
Determination of total arsenic *2*
Extraction of arsenic species 3
HPLC-ICPMS analyses 3
TURKEY PRODUCTION DATA **5**
Table S1. 2014 Turkey production data, by producer *5*
RESULTS **6**
Table S2. Pearson's correlation matrix for arsenic species in turkey meat samples (n=184) *6*

DETAILED LABORATORY METHODS SECTION

Reagents, standards and reference materials

Purified water (18.2 MΩ*cm, Millipore GmbH) was used throughout the analyses; for the digestion, nitric acid (> 69 % p.a.) from Roth was used following further purification in a quartz sub-boiling distillation unit; a germanium standard P/N 4400-1000201, Ge in 2 % nitiric acid, 1000 ± 3 µg Ge mL^{-1} (CPI International) was used as internal standard. A Single- Element Arsenic Standard P/N 4400-100031 (CPI International) As in 2 % nitric acid, 1000 ± 3 µg As mL^{-1}, was used for total arsenic measurement as an external calibration standard. For the water bath extraction, ammonium bicarbonate (≥ 99,0 % purum p.a., Sigma Aldrich), aqueous ammonia (≥ 25 % p.a., Roth) and hydrogen peroxide (30 % p.a., Roth) were used.

For arsenic speciation analyses, stock solutions containing 1000 mg $As{\cdot}L^{-1}$ as each of the following arsenic species were prepared in water or 1 % aqueous ammonia solution (for roxarsone and nitarsone only): arsenate prepared from $Na_2HAsO_4{\cdot}7H_2O$ (Merck); methylarsonate prepared in-house from As_2O_3 and CH_3I by the Meyer reaction; dimethylarsinate prepared from sodium dimethylarsinate (Fluka); and roxarsone (4-hydroxy-3-nitrophenyl) arsonic acid, grade Vetranal) and n*itarsone* (4-nitrophenyl arsonic acid) were purchased from Sigma Aldrich.

Instrumentation

ICPMS measurements were performed with an Agilent 7900, and HPLC was carried out with an Agilent 1100 series instrument (Agilent Technologies). The ICPMS was equipped with a concentric Micro Mist nebulizer and a Scott double pass spray chamber. The HPLC was equipped with a binary pump, a vacuum degasser, column oven, and an autosampler with a variable 100 µL injection loop; it was connected to the ICPMS with 0.125 mm PEEK (polyetheretherketone) tubing (Upchurch Scientific). Microwave digestions were performed with an UltraCLAVE III (MLS GmbH). Centrifugation was performed with a Scilogex D3024R (Scilogex) or a Rotina 420R centrifuge (Andreas Hettich GmbH & Co.KG).

Determination of total arsenic

Each sample was analysed for total arsenic content in duplicate (n=2) in the following manner. Portions (about 250 mg weighed with a precision of 0.1 mg) of the freeze-dried

powdered samples were weighed directly into 12 mL quartz tubes, and nitric acid (2 mL) and internal standard (100 µg L^{-1} Ge in 1 % nitric acid) was added. The tubes were transferred to a Teflon® rack of the UltraCLAVE microwave system and covered with Teflon® caps. After closing the system, an argon pressure of 4 x 10^6 Pa was applied and the mixture was heated to 250 °C for 30 minutes before being allowed to cool to room temperature. After mineralization, the samples were transferred to 15 mL polypropylene tubes (Greiner, Bio-one) and diluted with water to 10 mL. Standards for total arsenic determinations were prepared with 20 % nitric acid and 20 µg L^{-1} Ge for matrix matching with the digested samples. The digested samples were determined by ICPMS using helium as collision cell gas for minimizing polyatomic interferences from argon chloride ($^{40}Ar^{35}Cl$ on ^{75}As), and an optional gas (1 % CO_2 in argon) was used to enhance the arsenic response; a ten point calibration curve in the range 0.01 – 20 µg As L^{-1} was made for external calibration. The signal for As was normalized against that for the internal standard Ge to compensate for matrix effects.

Extraction of arsenic species

About 500 mg of each freeze-dried and homogenized samples were weighed into a 50 mL polypropylene tube (Greiner, Bio-one) and a solution (10 mL) of 20 mM ammonium bicarbonate adjusted to pH 9.2 with aqueous ammonia containing 1 % (v/v) hydrogen peroxide solution (30 % v/v) was added. The addition of hydrogen peroxide is necessary to convert all arsenite to arsenate. Samples were extracted by placing the tubes in a GFL-1083 shaking water bath (Gesellschaft für Labortechnik) at 60 °C for 60 minutes. After cooling to room temperature, the extracts were centrifuged for 15 min at 4700 rcf. The supernatant was filtered through a PTFE syringe filter (0.2 µm) (Bruckner Analysentechnik) before injection onto the HPLC system. Each sample was extracted in duplicate (n=2). When the values for arsenic species in the duplicates differed by more than 20%, the sample was re-analyzed.

HPLC-ICPMS analyses

Chromatographic separations were performed using a Dionex Ionpac® AS14A 150 mm x 3 mm; 5 µm particle size (Thermo Scientific, Switzerland) and a mobile phase consisting of ammonium bicarbonate (20 – 200 mM), adjusted to pH 9.2 with aqueous ammonia at a flow rate of 0.7 mL min^{-1}. The column temperature was 40 °C, injection volume 20 µL. The

following gradient of ammonium bicarbonate buffer was applied: 0-2 min: 20 mM; 2-4 min: 20 mM – 200 mM; 4-7 min: 200 mM; 7-7.1: 200 mM – 20 mM; 7.1-10 min: 20 mM.

An optional gas (1 % CO_2 in argon) was introduced through a T-piece connecting the spray chamber and the torch to enhance the arsenic response. In addition to the signal m/z 75 (^{75}As, $^{40}Ar^{35}Cl$) the signal at m/z 77 ($^{40}Ar^{37}Cl$) was monitored to check for possible chloride interference on m/z 75. The quantification was done by external calibration against standard arsenic species based on peak areas (calibration from 0.02 – 20 µg As·L^{-1}). For the quality control we used the standard reference material ERM-BC211 with certified contents of DMA (119 ± 13 µg As kg^{-1}) and iAs (124 ± 11 µg As kg^{-1}); we obtained 116 ± 4 µg As kg^{-1} for DMA and 99 ± 4 µg As kg^{-1} for iAs (n=9).

TURKEY PRODUCTION DATA

Table S1. 2014 Turkey production data, by producer

Producer code	2014 production (million kg)
Producer A	589.7
Producer E	580.6
Producer B	453.6
Producer K	224.1
Producer L	144.2
Producer F	127.0
Producer M	127.0
Producer N	113.4
Producer D	112.0
Producer O	106.1
Producer P	100.2
Producer Q	94.3
Producer R	90.7
Producer S	74.8
Producer G	68.0
Producer T	63.5
Producer U	43.1
Producer H	30.8
Producer V	20.4
Producer W	13.6
Producer C	11.3
Producer X	7.7
Producer Y	5.0

Production data are from National Turkey Federation, 2016. Producers A - H were represented in our sample (Producers I and J were smaller producers included in our sample for whom market share data were not available). Producers K – Y were not represented in our sample.

RESULTS

Table S2. Pearson's correlation matrix for arsenic species in turkey meat samples (n=184)

	Total As	iAs	MA	DMA	Nitarsone
Total As	1.00	-	-	-	-
iAs	0.77	1.00	-	-	-
MA	0.99	0.73	1.00	-	-
DMA	0.75	0.54	0.74	1.00	-
Nitarsone	0.95	0.79	0.93	0.70	1.00

Sample-specific concentration estimates used to estimate the correlation matrix were derived by taking the average of the replicate measurements from the same package (2 - 3 replicates for total arsenic and each arsenic species). For total arsenic, iAs, MA and DMA measurements below the detection limit, we imputed the value of the detection limit divided by the square root of two. For nitarsone, samples below the detection limit were given the value of 0.
